# Two-dimensional temperature-responsive chromatography using a poly(*N*-isopropylacrylamide) brush-modified stationary phase for effective therapeutic drug monitoring

**DOI:** 10.1038/s41598-022-06638-1

**Published:** 2022-02-16

**Authors:** Kenichi Nagase, So Inoue, Masakazu Inoue, Hideko Kanazawa

**Affiliations:** Faculty of Pharmacy, Keio University, 1-5-30 Shibakoen, Minato, Tokyo 105-8512 Japan

**Keywords:** Analytical chemistry, Green chemistry

## Abstract

Therapeutic drug monitoring (TDM) is an effective pharmacological approach for controlling drug concentration in a patient’s serum. Herein, a new two-dimensional chromatography system was developed using two poly(*N*-isopropylacrylamide) (PNIPAAm)-modified bead-packed columns for effective and safe drug monitoring. PNIPAAm-modified silica beads were prepared as packing materials using atom transfer radical polymerization of NIPAAm. The increase in the retention times of the drugs requiring TDM with increasing temperature, was attributed to enhanced hydrophobic interactions at elevated temperatures. The drugs and serum proteins were separated on the prepared column at 40 °C using an all-aqueous mobile phase. Differences in the hydrophobic interactions accounted for the elution of the serum proteins and drugs at short and long retention times, respectively, and a primary column was employed to separate the serum proteins and drugs. After eluting the serum proteins from the column, the drug was introduced into the secondary column, leading to a peak of its purified form and enabling determination of the drug concentration. Two-dimensional temperature-responsive chromatography can benefit TDM by allowing the drug concentration in the serum to be measured in all-aqueous mobile phases without sample preparation.

## Introduction

Therapeutic drug monitoring (TDM), that is, the monitoring of drug concentrations in patients, is essential for effective pharmacological therapy, as the dosages of specific types of drugs differ among patients because of their varying absorption, distribution, and elimination levels^1^. Thus, the dosage must be adjusted based on the drug concentration in the patient’s serum; a low drug concentration may be ineffective, whereas an excessive concentration may be toxic^2,3^. Various methods including immunological and chromatographic methods have been developed for TDM^4^. Liquid chromatography (LC) is an effective and versatile method for monitoring drug concentrations in the serum because it does not require a specific drug ligand^5^. In contrast, ordinary chromatography requires an organic solvent in the mobile phase to adjust drug retention in the column. However, it is challenging to use organic solvents in hospitals because of exposure risks. Additionally, ordinary chromatography requires sample preparation (pretreatment) to remove serum proteins. This time-consuming sample preparation process, which generally requires organic solvents for serum protein denaturation, delays the monitoring of drug concentrations in a patient.

To overcome these issues, we developed a method based on temperature-responsive chromatography using poly(*N*-isopropylacrylamide) (PNIPAAm)-modified bead-packed columns and two-dimensional chromatography. Temperature-responsive chromatography employing a PNIPAAm-modified stationary phase can modulate analyte retention by changing the column temperature through the intrinsic properties of PNIPAAm^6–8^. Various types of thermoresponsive polymers, including poly(*N*-alkyl acrylamide)s, poly(alkyloxide) copolymers, and poly(2-alklyl-2-oxazoline)s, have been investigated^9^. Among these, PNIPAAm is the most widely utilized because of its sharp phase-transition properties^10–12^. PNIPAAm exhibits temperature-dependent hydrophilic and hydrophobic changes, which can be attributed to hydration and dehydration processes across its phase-transition temperature of 32 °C^10,13^. In addition, PNIPAAm exhibits expansion and shrinkage at low and high temperatures, respectively. Thus, different biomedical applications, such as temperature-modulated drug and gene delivery systems^14–19^, biosensor and bioimaging systems based on the phase-transition of PNIPAAm^20–25^, nano-actuators induced by chemical oscillations^26–28^, cell separation systems exploiting the difference between the adhesive properties of cells to PNIPAAm^29–32^, and cell culture substrates for tissue engineering^33–39^, have been developed based on the thermoresponsive properties of PNIPAAm. A PNIPAAm-modified stationary phase is generally used for chromatography. Because the hydrophobicity of PNIPAAm changes with changing column temperature, the hydrophobic interactions between PNIPAAm and analytes can be modulated to control analyte retention in the columns. Thus, chromatography does not require addition of an organic solvent to the mobile phase to control analyte retention. The column performance of the analytes in the PNIPAAm-modified stationary phase was determined from the amount of PNIPAAm in the stationary phase^8^. Studies have reported that a PNIPAAm brush-modified stationary phase, which was prepared via atom transfer radical polymerization (ATRP), exhibited good performance because of the precisely controlled graft density and chain length of PNIPAAm^40^.

Two-dimensional chromatography is an effective approach for LC^41,42^. The chromatography system is based on two columns and a switching valve. The first column (primary column) separated the analyte, and the effluent from the primary column containing the measuring object was introduced into the second column (secondary column) by switching the valve at an appropriate time. In the two-dimensional chromatography system, the primary column is employed for sample preparation, and the secondary column is employed for subsequent measurements.

**Figure 1:**
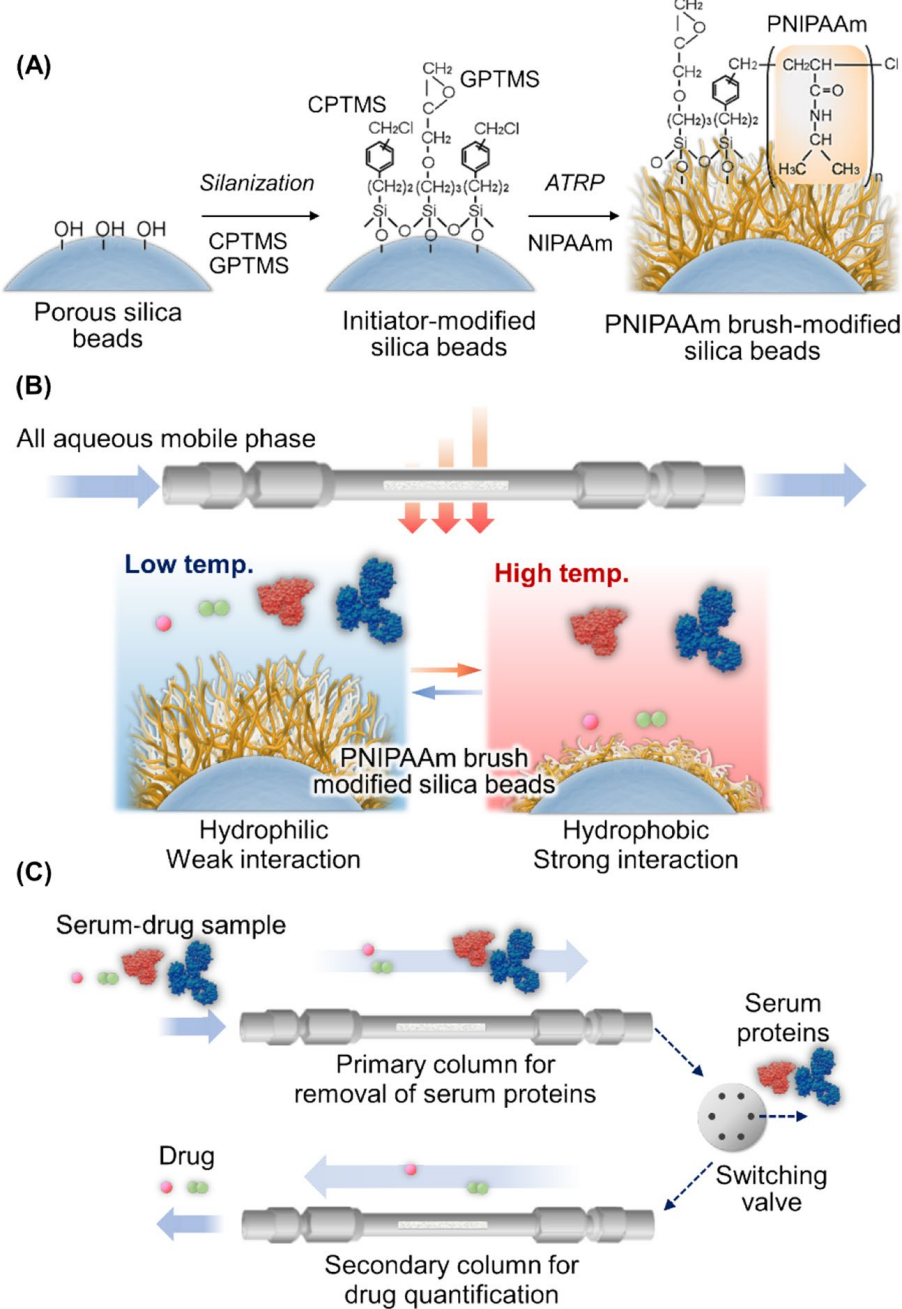
Temperature-responsive chromatography for therapeutic drug monitoring. (**A**) Schematic illustration of the preparation of thermoresponsive polymer-modified beads as packing materials via silanization and ATRP. (**B**) Interaction between the drug sample and analyte. (**C**) Two-dimensional temperature-responsive chromatography of the serum–drug sample.

In this study, a two-dimensional chromatography system consisting of two PNIPAAm brush-modified bead-packed columns was developed (Fig. 1). PNIPAAm brush-modified silica beads were prepared as packing materials using ATRP. Thereafter, the prepared silica bead-packed columns were used in a two-dimensional chromatography system. Drugs in the serum proteins were analyzed to investigate the applicability of the developed system for TDM.

## Results and discussion

### Characterization of polymer-modified silica beads

The PNIPAAm brush-modified silica beads were prepared in a silane coupling reaction of ((chloromethyl)phenylethyl)trimethoxysilane (CPTMS) with glycidyloxypropyltrimethoxysilane (GPTMS) to form silica beads, followed by ATRP of NIPAAm (Fig. 1A). We developed a functional PNIPAAm-modified stationary phase for TDM. A PNIPAAm brush with a low polymer density was prepared on silica beads by diluting CPTMS (ATRP initiator) on the beads, as the highly dense PNIPAAm brush-modified bead-packed column exhibited an excessively long retention time for analytes in previous studies^40,43^; this long retention time was not suitable for the short analysis time required for TDM.

The prepared beads were characterized using elemental analysis, attenuated total reflection Fourier-transform infrared spectroscopy (ATR/FT–IR), and scanning electron microscopy (SEM). Elemental analysis of the prepared beads at each reaction step was performed to investigate the amounts of immobilized silane and modified PNIPAAm (Table 1). The carbon composition of silane-layer-modified beads was higher than that of unmodified silica beads, indicating the success of the silane coupling reaction under the reaction conditions used in this study. The thickness of the modified silane layer was 1.65 μmol/m^2^, which is relatively small compared to that of beads solely modified with CPTMS, as previously reported (~ 5.0 μmol/m^2^)^44^; this is because the molecular size of GPTMS is slightly larger than that of CPTMS, thus lowering the immobilized density of the silane layer. The PNIPAAm brush-modified beads (PN-1000 and PN-1500) exhibited larger carbon compositions compared to the silane layer-modified silica beads, indicating successful modification of PNIPAAm on the silica beads via ATRP under the conditions employed in this study. The amounts of PNIPAAm, which were modified, were 2.39 and 2.65 mg/m^2^ for PN-1000 and PN-1500, respectively. A relatively larger amount of PNIPAAm was observed in PN-1500 than in PN-1000 because the high monomer concentration (1500 mM) in ATRP increased the polymerization rate compared to the lower monomer concentration (1000 mM).

**Table 1:** Characterization of the temperature-responsive hydrogel-modified beads employing CHN modified beads.

Code	Monomer concentration in ATRP (mmol/L)	Carbon composition (%)	%C_(calcd.)_	Immobilized silane (μmol/m^2^)	Grafted polymer (mg/m^2^)
Unmodified silica beads	–	0.54			
Initiator modified silica (CPTMS:GPTMS = 75:25)	–	2.13	57.2	1.65	
Short PNIPAAm-modified silica beads (PN-1000)	1000	14.0	63.7		2.39
Long PNIPAAm-modified silica beads (PN-1500)	1500	15.1	63.7		2.65

Carbon composition was determined using CHN elemental analysis, in which %C_(calcd.)_ was calculated as the percentages of the molecular weight of carbon in the initiator and copolymer. The amounts of initiator and polymer on the silica beads were estimated from the carbon composition.

**Figure 2:**
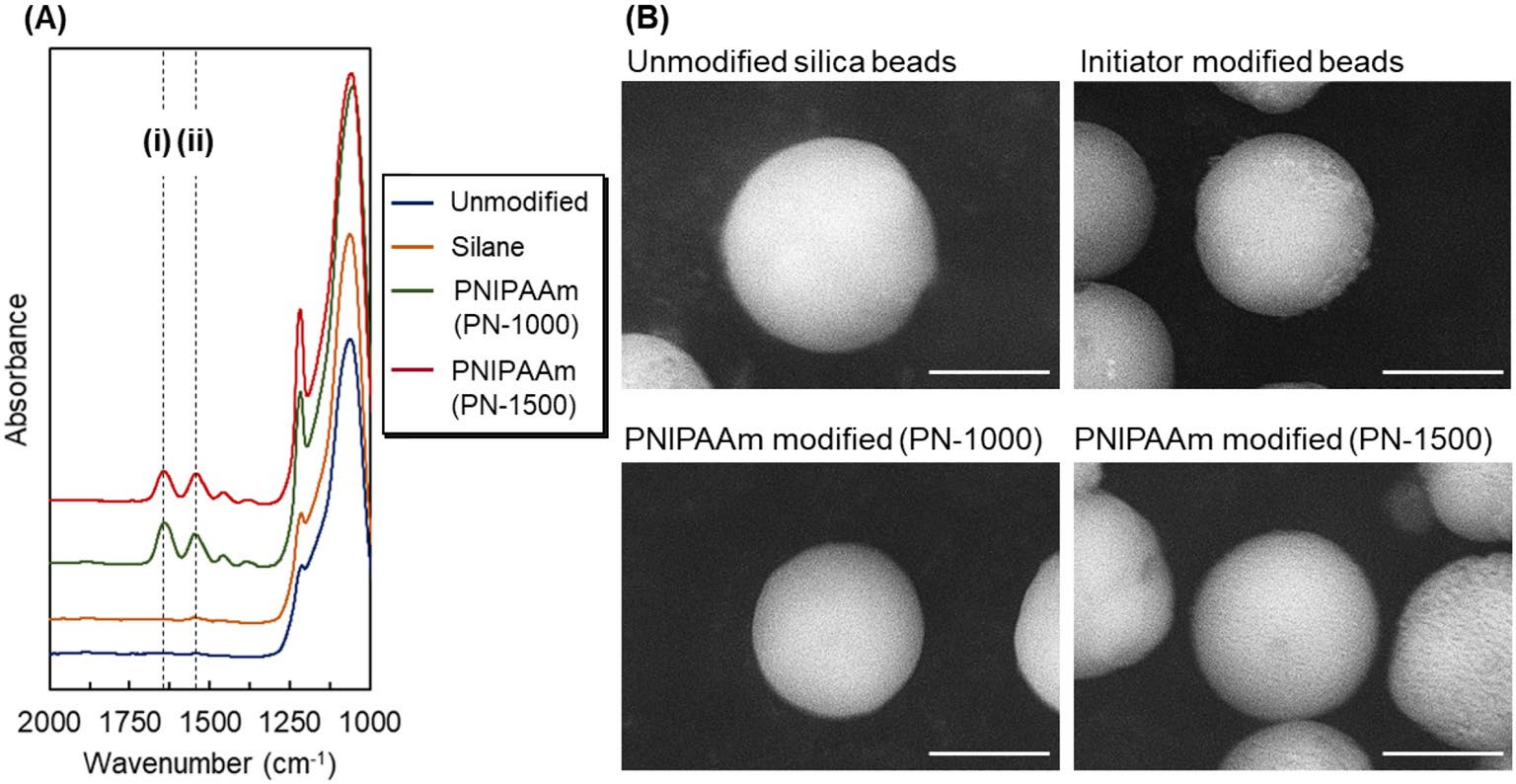
Characterizations of the prepared beads. (**A**) Fourier-transform infrared spectra of the prepared beads. The dashed lines, (i) and (ii), indicate the peak attributed to C = O stretching and N–H bending, respectively. Each spectrum was offset to avoid overlapping each spectrum. (**B**) Scanning electron microscopy images of the prepared beads. Scale bars: 3 μm.

At each reaction step, the prepared beads were characterized using ATR/FT–IR spectroscopy (Fig. 2A). The baseline of each spectrum was offset to describe the peak shapes clearly. A large peak was observed at 1060 cm^−1^, which was attributed to the Si–O bond of the silica beads. Two peaks were observed at 1550 and 1645 cm^−1^ for PN-1000 and PN-1500, respectively, whereas no peaks were observed at these wavenumbers for unmodified silica and silane-modified beads. These peaks were attributed to the N–H and C=O bonds of the amido group of PNIPAAm. Thus, these results indicate the modification of PNIPAAm on silica beads via ATRP. In addition, a previous report showed that the peak intensity of the amide bond represents the amount of modified PNIPAAm^45,46^. Because the amount of PNIPAAm on PN-1500 was larger than that on PN-1000, the intensity of the amide bond of PN-1500 was expected be larger than that of PN-1000. However, the heights of these peaks were approximately the same. This is likely attributed to measurement error of the ATR/FT–IR. In the measurement, the absorbance of the samples changed slightly depending on their contact with the ATR crystal. Thus, no differences in the peak height were observed between PN-1000 and PN-1500.

SEM performed at each reaction step (Fig. 2B) revealed that the beads had similar spherical morphologies, indicating that the silane coupling reaction and ATRP did not deform the silica beads. Additionally, bead aggregation was not observed after ATRP, indicating that the polymerization reaction was controlled by PN-1000 and PN-1500.

### Evaluation of column performance

The prepared PNIPAAm brush-modified silica beads were packed into a stainless steel column (internal diameter = 2.1 mm × length = 50 mm or 100 mm). To investigate column performance, the elution behavior of steroids from the column was observed at various temperatures (Supplementary Fig. S1); the properties of the steroids are summarized in Supplementary Table S1. The retention time of the steroids on the columns increased, and the mixture of steroids was separated with increasing column temperature because PNIPAAm on the silica beads was dehydrated and became hydrophobic with increasing temperature, during which, hydrophobic interactions between PNIPAAm and the steroids increased. Additionally, PN-1500 exhibited a longer retention time compared to PN-1000 because the larger amount of PNIPAAm on PN-1500 than on PN-1000 induced strong hydrophobic interactions with the steroids. These results indicate that PN-1500 is suitable for relatively hydrophilic analytes because they require strong hydrophobic interactions to achieve retention in the column. In contrast, PN-1000 is suitable for relatively hydrophobic analytes. Hydrophobic analytes exhibit long retention times because of their high hydrophobicity. Thus, PN-1000 exhibited a relatively short retention time, rendering it suitable for hydrophobic analytes and short analysis times.

### Elution behaviors of drugs in therapeutic drug monitoring

**Figure 3:**
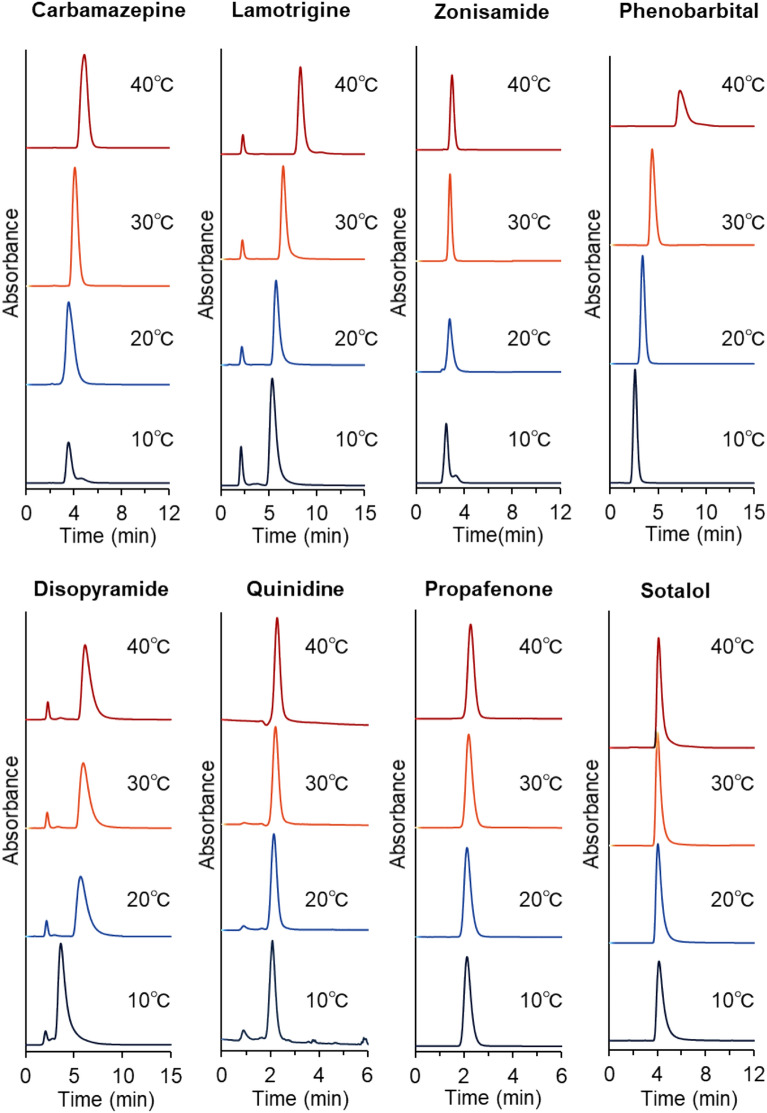
Temperature-dependent elution behaviors of the antiepileptic and antiarrhythmic drugs from the PNIPAAm brush-modified bead-packed column. Columns: PN-1000 packed beads, 2.1 mm internal diameter × 50 mm for carbamazepine; PN-1000 packed beads, 2.1 mm internal diameter × 100 mm for zonisamide; PN-1500 packed beads, 2.1 mm internal diameter × 100 mm for the other drugs. Mobile phase: 10 mM CH_3_COONH_4_ (pH 6.75) at a flow rate of 0.2 mL/min. The detection of each drug is summarized in Table S2.

**Figure 4:**
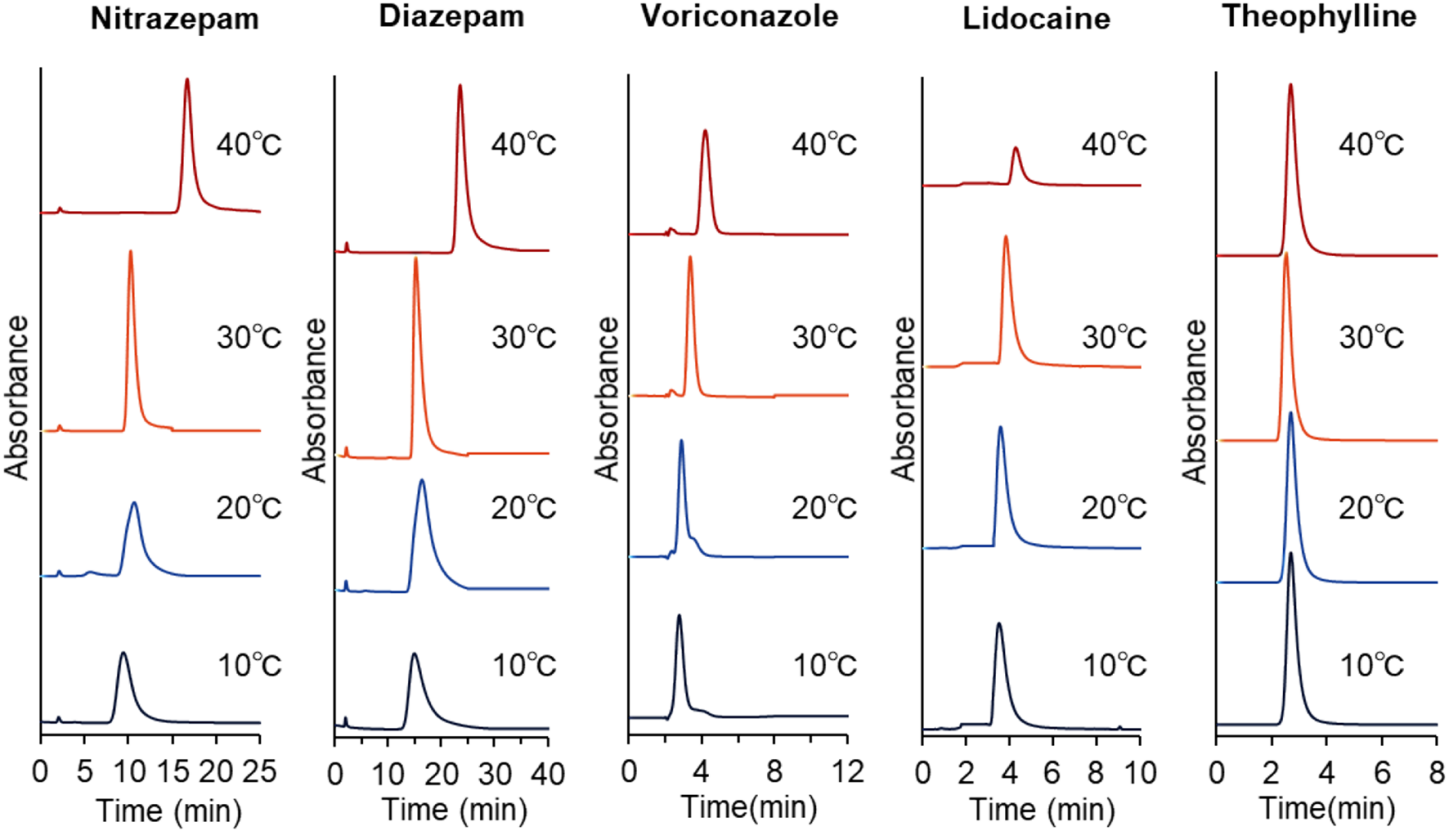
Temperature-dependent elution behavior of drugs utilized in therapeutic drug monitoring from the PNIPAAm brush-modified bead-packed columns. Columns: PN-1000 packed beads, 2.1 mm internal diameter × 100 mm for voriconazole; PN-1500 packed beads, 2.1 mm internal diameter × 100 mm for all other drugs. Mobile phase: 10 mM CH_3_COONH_4_ (pH 6.75) at a flow rate of 0.2 mL/min. The detection of each drug is summarized in Table S2.

To investigate the performance of the prepared PNIPAAm brush-modified bead-packed columns in the analysis of drugs for TDM, the elution behaviors of 13 drugs requiring TDM from the columns were observed (Figs. 3, 4); the properties of these drugs are summarized in Supplementary Table S2. The elution behaviors of the antiepileptic and antiarrhythmic drugs are summarized in Fig. 3. The retention times of most drugs increased with increasing column temperatures because dehydration of PNIPAAm increased the hydrophobic interactions between PNIPAAm and the drugs. The increases in the retention times of quinidine and propafenone were smaller than those of the other drugs because of the hydrophilicity of quinidine and propafenone, which weakened the interactions between dehydrated PNIPAAm and the drugs. The retention time of sotalol slightly decreased with increasing temperature. This was likely because of the increased solubility of sotalol in the mobile phase with increasing temperature. In temperature-responsive chromatography, the hydrophobic interaction between the PNIPAAm-modified stationary phase and analyte increased with increasing column temperature, attributed to dehydration of PNIPAAm, which led to increased retention times. In contrast, analyte solubility in the mobile phase increased with increasing temperatures, leading to a decreased retention time. Thus, the analyte retention time was determined from the balance of these factors. For sotalol, the effect of the increase in solubility in the mobile phase was large compared with the increase in the hydrophobic interaction between the analyte and PNIPAAm.

The lower column temperatures exhibited wider peaks compared to higher column temperatures, except for phenobarbital because the PNIPAAm on the silica beads shrank at high temperatures, thereby preventing analyte diffusion into the PNIPAAm brush layer. For phenobarbital, a large peak width was observed at 40 °C. Additionally, a large increase in the retention time of phenobarbital was observed between 30 and 40 °C. The specific retention time change of phenobarbital may be related to its structure. Phenobarbital contains two amide bonds (Supplementary Table S2), and PNIPAAm also has amide bonds. Thus, phenobarbital interacts with PNIPAAm through hydrogen bonding as compared to other drugs. Phenobarbital has a benzene ring adjacent to the amide bond, which interacts with PNIPAAm through hydrophobic interactions in addition to hydrogen bonding. In a previous report on temperature-responsive chromatography, phenobarbital exhibited a relatively longer retention time compared to other types of barbiturates with similar structures, barbital and allobarbital^47^. This also indicated that the phenobarbital structure caused specific retention on the PNIPAAm column. Additionally, disopyramide exhibited specific retention profiles, likely because of the amide and isopropyl groups of the disopyramide molecule. These functional groups in disopyramide may interact with the amide and isopropyl groups of PNIPAAm, leading to unique retention profiles on the PNIPAAm column. All drugs were eluted within 10 min from the column, indicating that the analysis can be performed in a short time.

The elution behavior of the five other drugs from the prepared column was observed (Fig. 4). The retention times of these drugs also increased as the column temperature increased because dehydration of PNIPAAm, as well as hydrophobic interactions, were enhanced at higher temperatures. Nitrazepam and diazepam exhibited large increases in their retention times between 30 and 40 °C, likely because the phase-transition temperature of PNIPAAm is 32 °C, and hydrophobic interactions between PNIPAAm and the drugs largely increased with temperature. The peaks of nitrazepam and diazepam at lower temperatures (10 °C and 20 °C) were relatively wide, reducing the quantity at low drug concentrations because of the difficulty in estimating the peak area. The wider peaks were attributed to diffusion of these drugs into the PNIPAAm brush layers at lower temperatures. In contrast, sharp peaks were observed at high temperatures of 30 °C and 40 °C because drug diffusion into the brush layers was suppressed by shrinking PNIPAAm. Thus, sharp peaks were observed at high column temperatures, which is favorable for peak area determination even at low drug concentrations.

**Figure 5:**
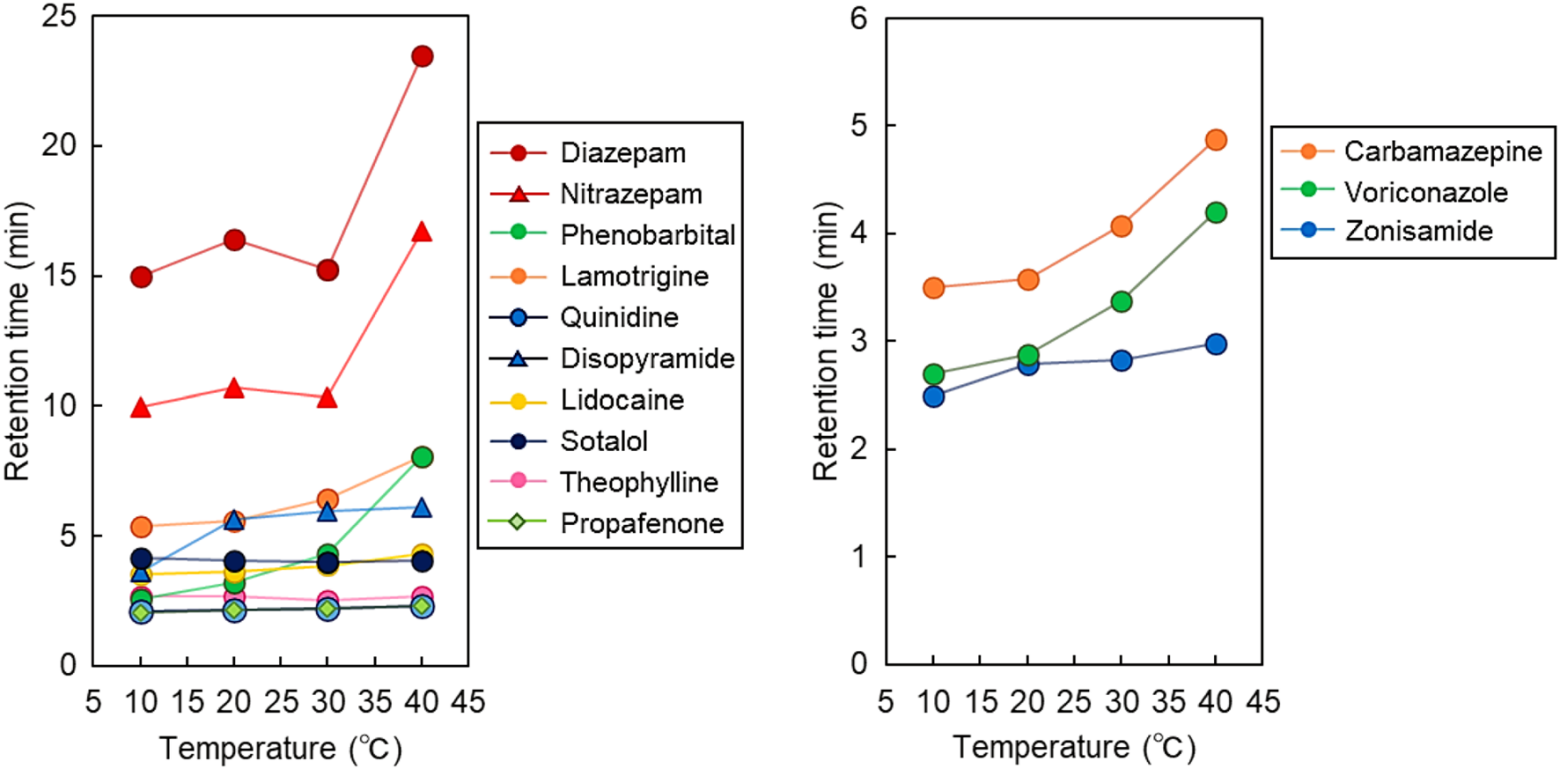
Retention times of drugs utilized for therapeutic drug monitoring employing the PNIPAAm brush-modified bead column.

To compare the temperature dependence of the retention time of each drug, the relationships between their retention times and column temperatures were plotted (Fig. 5). The retention times of most drugs increased with increasing column temperatures and increased significantly between 30 and 40 °C because of dehydration of PNIPAAm with increasing temperatures. The retention times of sotalol, theophylline, and propafenone were independent of column temperature, likely because of the low hydrophobicity of the drugs, which led to inadequate hydrophobic interactions between PNIPAAm and the drugs for column retention. To investigate the repeatability of the prepared column, the average retention times of the drugs, following three repeated measurements in each case, were determined; their relative standard deviations are summarized in Supplementary Table S3, and the relative standard deviations for all drugs were quite small (< 0.3%) at all column temperatures. Thus, the prepared columns exhibited repeatability for drug measurement.

### Elution behavior of serum–drug sample

**Figure 6:**
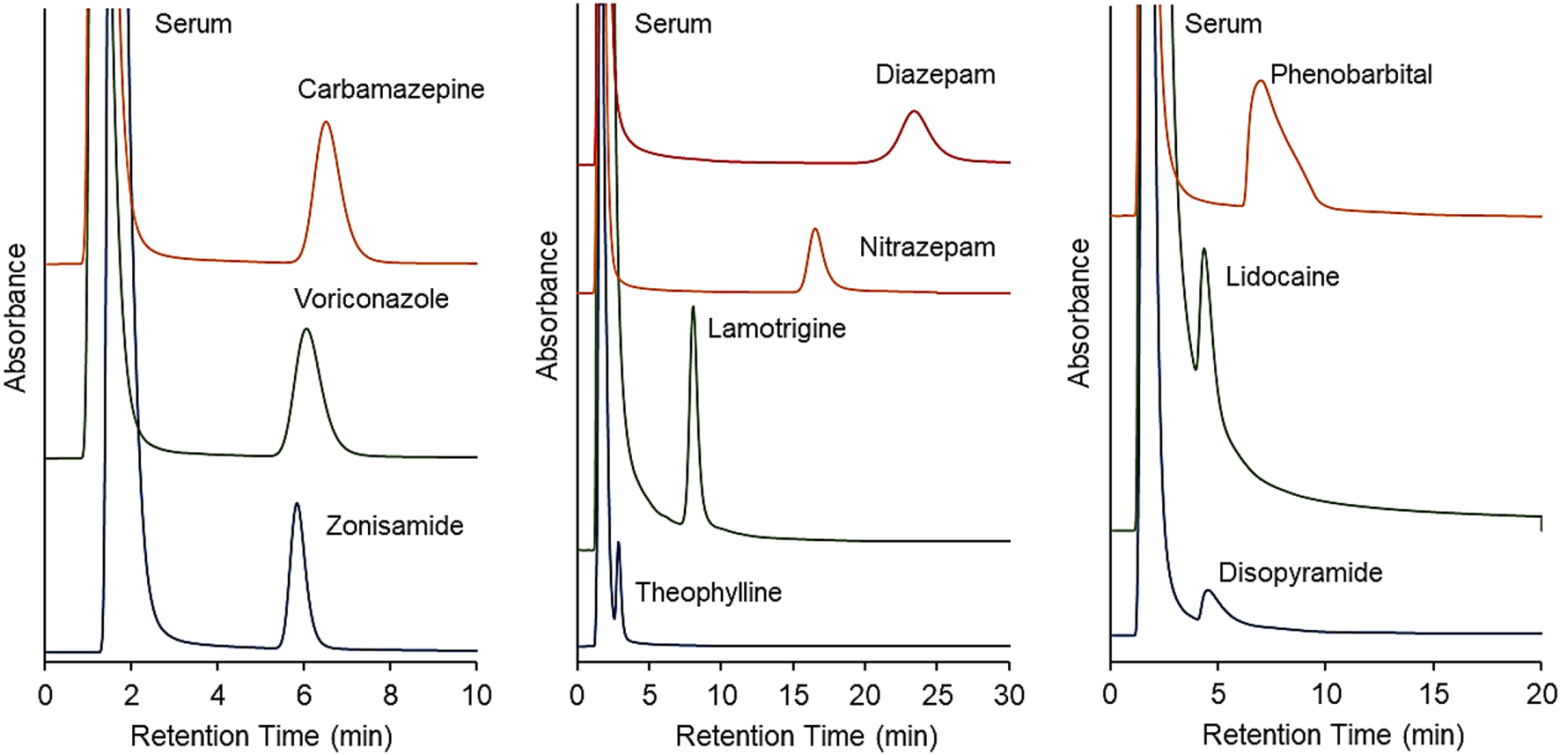
Chromatograms of drugs with the serum proteins in PNIPAAm brush-modified bead-packed columns. Columns: PN-1500 packed beads, 2.1 mm internal diameter × 50 mm for carbamazepine and voriconazole; and PN-1500 packed beads, 2.1 mm internal diameter × 100 mm for all other drugs. Mobile phase: 10 mM CH_3_COONH_4_ (pH 6.75) at a flow rate of 0.2 mL/min. Column temperature: 40 °C. The detection wavelength of each drug is summarized in Table S2.

Under normal conditions, TDM is generally performed by measuring drug concentration in serum. In reversed-phase LC, a serum sample must be deproteinated before it is injected into a high-performance LC (HPLC) column to prevent the adsorption of serum proteins, which can cause column clogging, onto the beads in the column. In contrast, previous reports revealed that PNIPAAm copolymers without ionic group-modified bead-packed columns eluted proteins without column clogging because non-ionic PNIPAAm copolymers did not show adsorption of  the proteins^48–55^. Thus, the elution behavior of the serum–drug samples was observed on the prepared bead-packed column (Fig. 6). The column temperature was maintained at 40 °C, at which, effective retention of all drugs with a sharp peak was observed. The chromatograms of all serum–drug samples, except those of theophylline and lidocaine, revealed that the serum proteins were eluted from the column within short retention times, whereas the drugs were eluted during subsequent periods, indicating separation of the serum proteins and drugs, and that the drug concentrations could be determined for all samples. The peaks of theophylline and lidocaine slightly overlapped with those of the serum proteins, likely because of the low hydrophobicity of these drugs and weak interaction between them and PNIPAAm, making it challenging to measure their concentrations in serum samples using the prepared column.

The average retention time and relative standard deviation of each drug were observed via three repeated measurements (Supplementary Table S4), and the relative standard deviations of their retention times were quite small (< 0.3%), although the drug sample contained serum proteins. These results indicate that the prepared columns exhibited repeatability for drug measurement, even in the presence of serum proteins.

### Removing serum proteins via two-dimensional chromatography

**Figure 7:**
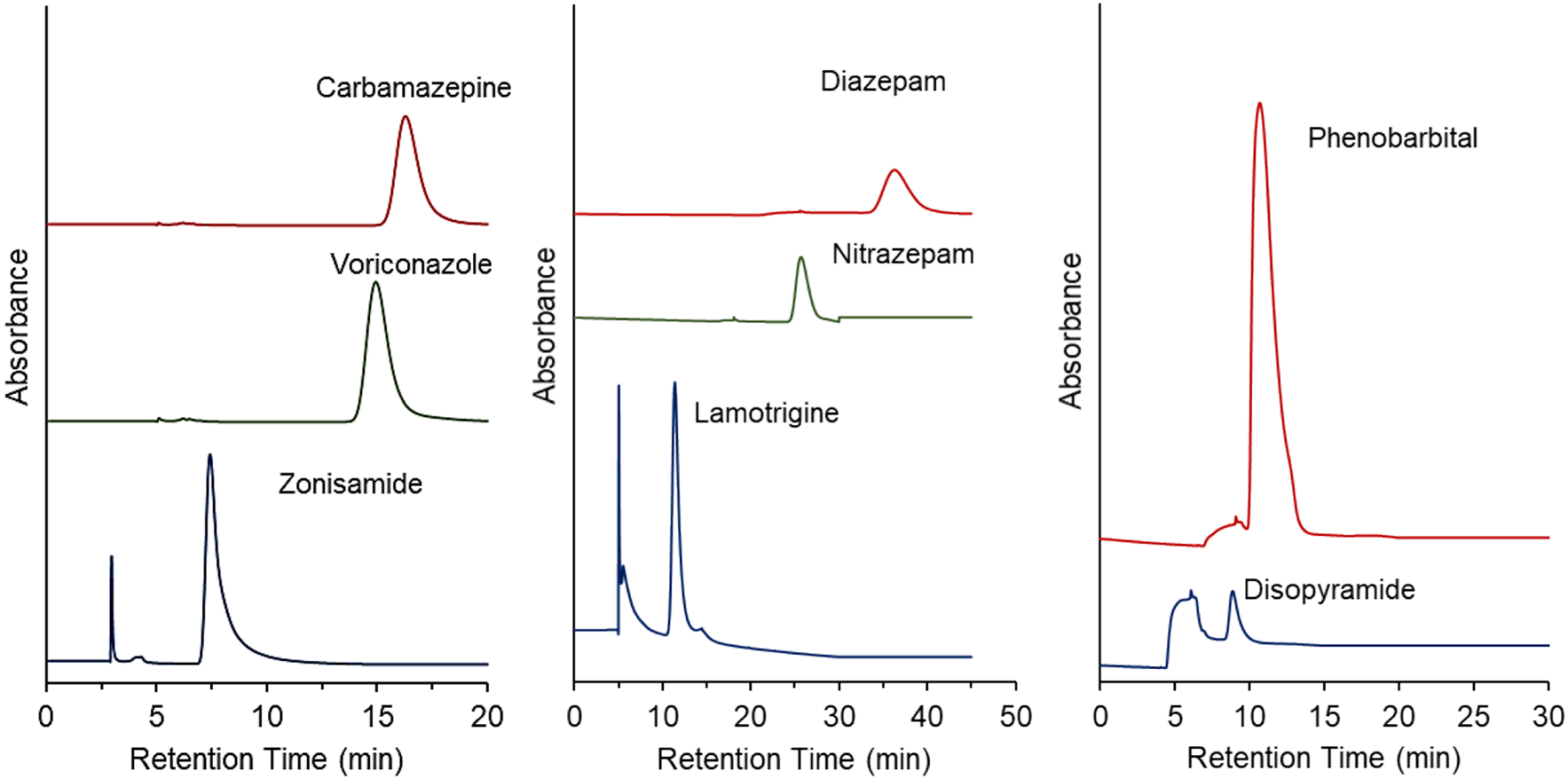
Chromatograms of drug sample in serum proteins after removing serum proteins via heart-cutting two-dimensional HPLC. Primary column: PN-1500 packed beads, 2.1 mm internal diameter × 50 mm for carbamazepine and voriconazole; PN-1500 packed beads, 2.1 mm internal diameter × 100 mm for all other drugs. Secondary column: PN-1500 packed beads, 2.1 mm internal diameter × 50 mm. Mobile phase: 10 mM CH_3_COONH_4_ (pH 6.75) at a flow rate of 0.2 mL/min. The detection wavelength of each drug is summarized in Table S2. The column-switching durations are summarized in Table S5.

Serum proteins and drugs were separated using the developed PNIPAAm brush-modified bead-packed column. However, conducting many measurements through repeated injection of serum proteins would gradually reduce the column performance and repeatability for measuring drug concentrations. Thus, a two-dimensional temperature-responsive chromatography system was developed for removing serum proteins from the primary column and measuring the drug concentration in the secondary column (Fig. 1C). The two prepared PNIPAAm brush-modified columns were connected to a two-dimensional HPLC system. The two columns were not connected initially. A drug-serum protein sample was injected into the system, and the serum and drug were separated through the primary column based on the differences in their retention in the column. Subsequently, the primary column was connected to a secondary column after eluting serum proteins from the primary column. The drug was allowed to flow to the secondary column. The chromatograms of drugs in the secondary column are shown in Fig. 7, and the times of connecting the columns are summarized in Supplementary Table S5. Furthermore, the serum proteins were eluted from the drugs in the primary column, and the purified drugs were observed in the chromatogram of the secondary column. Thus, the serum proteins were not introduced into the secondary column, improving the accuracy of the measurement of the drug concentration and preventing the column from deterioration. Very small amounts of proteins were observed as contaminant peaks in the chromatograms of zonisamide, lamotrigine, and disopyramide, possibly because of the introduction of slightly eluted serum proteins from the primary column into the secondary column. In the chromatogram, a wider peak was observed compared to that in the chromatogram of the drug without serum (Figs. 3, 4). This was because of the increased column length when using a two-dimensional chromatography system. In analysis of the elution behavior of each drug without serum (Figs. 3, 4), the drug was eluted from one column, resulting in relatively sharp peaks. In contrast, in the two-dimensional chromatography system, the drug flowed through two serially connected columns, leading to a wider peak and slight peak tailing.

The average retention time and relative standard deviation of each drug were determined from three repeated measurements (Supplementary Table S6). The relative standard deviation of the retention times was relatively small (< 0.2%), even when a two-dimensional HPLC system was employed. These results indicate that the constructed two-dimensional chromatography system exhibited sufficient repeatability for measuring drug concentrations. To investigate the quantitative ability of the proposed method, calibration curves of the drugs separated using a two-dimensional HPLC system were drawn (Supplementary Fig. S2). A linear relationship between the drug concentration and peak area was observed in the drug concentration range for TDM. Each calibration curve exhibited a high correlation coefficient (> 0.998), indicating that the drug concentration can be measured using the developed two-dimensional temperature-responsive chromatography system with an all-aqueous mobile phase and without a sample preparation process. Even at lowest concentration of each drug, which were 5.0 μg/mL zonisamide, 2.0 μg/mL carbamazepine, 2.0 μg/mL voriconazole, 3.0 μg/mL lamotrigine, and 5.0 μg/mL phenobarbital, the plots showed a linear approximation, indicating that the calibration curve can be used to estimate low concentrations.

**Figure 8:**
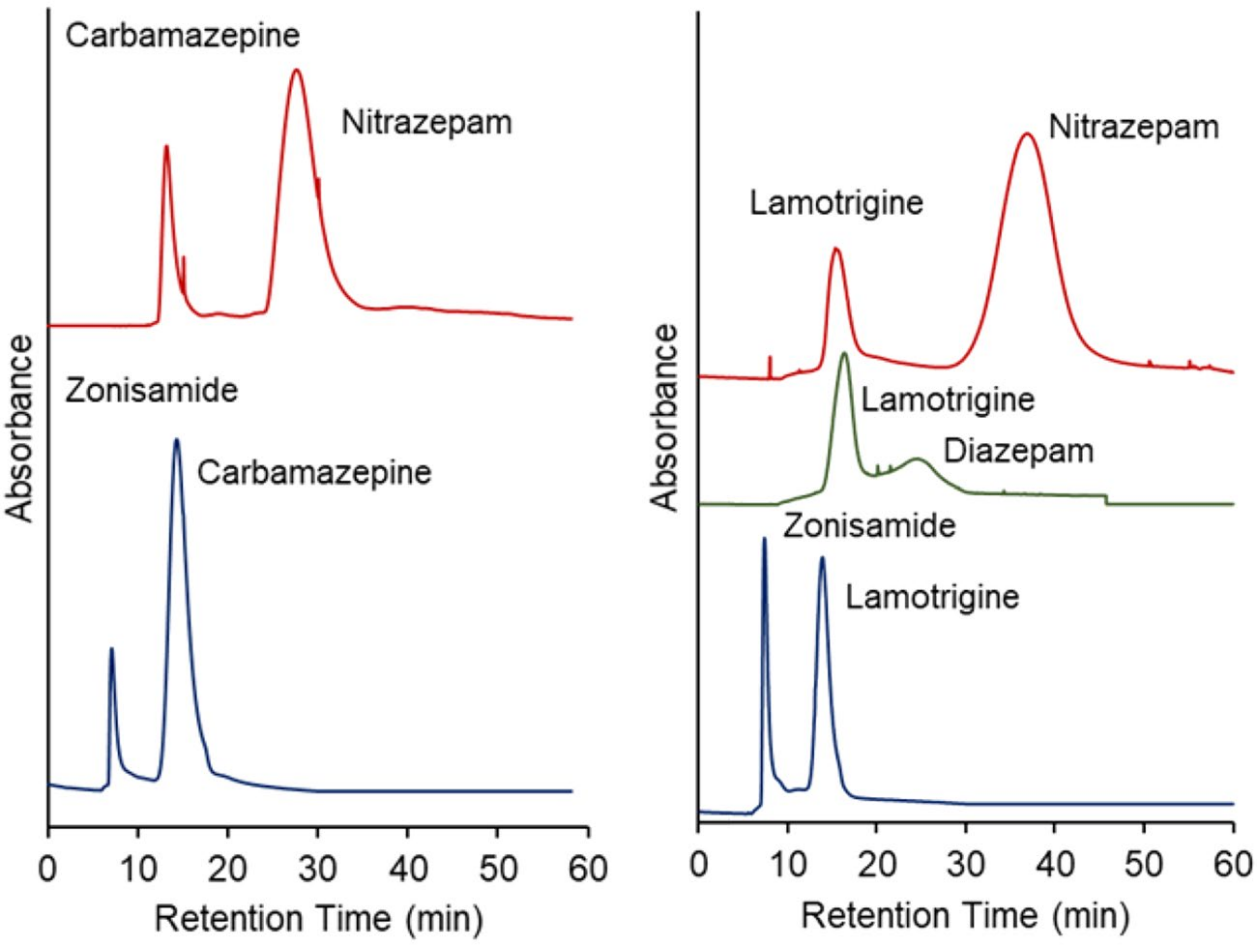
Chromatograms of drug mixtures with serum proteins via two-dimensional temperature-responsive chromatography. Primary column: PN-1500 packed beads, 2.1 mm internal diameter × 100 mm for all drugs. Secondary column: PN-1500 packed beads, 2.1 mm internal diameter × 50 mm. Mobile phase: 10 mM CH_3_COONH_4_ (pH 6.75) at a flow rate of 0.2 mL/min.

The separation of a mixture of drugs from serum proteins was investigated using the developed chromatography system. Monotherapy, which requires only one drug, is the main therapy when antiepileptic drugs are involved. However, a combination of antiepileptic drugs is sometimes administered to patients who do not respond to monotherapy^56^, and the concentrations of the two types of drugs in the serum must be determined. Thus, the suitability of the developed two-dimensional chromatography system for monitoring a mixture of two antiepileptic drugs was investigated. A mixture of two antiepileptic drugs in the serum was employed as the model sample, and their elution behaviors were observed using the two-dimensional chromatography system (Fig. 8). The time required to connect the columns is summarized in Supplementary Table S7. The serum protein was eluted using the primary column, and the two drugs were separated using the secondary column, as shown in the chromatogram. The average retention time and relative standard deviation of each drug were determined from three repeated measurements (Supplementary Table S8). The relative standard deviation of the retention times was small (< 0.4%), even when a two-dimensional HPLC system and drug mixture of drugs were used. Thus, the developed two-dimensional chromatography system exhibited sufficient repeatability for measuring a mixture of drugs in serum proteins.

In the case of polypharmacy, multiple drugs in the serum must be detected, and sharper peaks in the chromatogram are desired. To obtain sharper peaks in the chromatogram, temperature-responsive monolithic silica columns would be suitable as secondary columns, as previously reported temperature-responsive monolithic silica columns exhibited sharp peaks of analytes in the chromatogram compared to bead-packed columns^57–60^. In addition, the detection limit can be further improved by using a monolithic silica column as a secondary column, as a sharp peak in the chromatogram can provide a more accurate estimation of the peak area, improving drug detection.

The results indicate that the developed PNIPAAm brush-modified bead-packed column can be used to determine the drug concentrations of serum proteins by separating the serum proteins and drugs. Thus, a two-dimensional chromatography system comprising two columns (primary column for separating proteins from drugs and secondary column for monitoring drug concentration) was developed. The system can benefit TDM in medical settings because it does not require sample preparation (deproteinization) or the utilization of organic solvents as the mobile phase.

## Conclusions

A two-dimensional temperature-responsive chromatography system was developed to determine the drug concentration in a serum sample. PNIPAAm brush-modified silica beads were prepared using surface-initiated ATRP. These beads were packed into a stainless steel column, and the drug retention profiles of these columns were observed. The retention time increased with increasing column temperatures because of the enhanced hydrophobic interactions between PNIPAAm and the drugs. Drugs in the serum proteins were injected into the column, and the serum proteins and drugs were separated in the column based on differences in their hydrophobic interactions. A two-dimensional temperature-responsive chromatography system was developed using two PNIPAAm brush-modified bead-packed columns. The primary column was used to separate the serum proteins and drugs, after which the serum proteins were allowed to flow into the waste. Thereafter, the purified drug was introduced into the secondary column, in which only peaks of the purified drug were obtained. From the chromatogram, a calibration curve exhibiting a high correlation coefficient was obtained, ensuring accurate determination of the drug concentration of serum proteins. The results further indicate that the two-dimensional temperature-responsive chromatography system can benefit TDM by determining the drug concentration in serum without sample preparation (deproteination) and the utilization of organic solvents as the mobile phase.

## Methods

### Preparation of PNIPAAm brush-modified beads

As shown in Fig. 1A,PNIPAAm-modified silica beads, which were employed as chromatographic packing materials, were prepared as follows: silica beads (diameter: 5 μm pore diameter: 300 Å, 100 m^2^/g) were washed with hydrochloride for 3 h at 90 °C. The beads were then filtered, rinsed with pure water, and vacuum-dried for 15 h at 150 °C. The silica beads (6.2 g) were placed in a flask and humidified at relative humidity at 25 °C for 4 h. Two silane coupling reagents (CPTMS (1.25 mL, 4.97 mmol) and GPTMS (0.366 mL, 1.66 mmol)), were added to toluene (124 mL), and the solution was poured into a flask containing the humidified silica beads. The silane coupling reaction was performed for 16 h at 25 °C, after which the beads were washed with toluene and acetone and vacuum-dried for 3 h at 110 °C.

PNIPAAm was modified onto the silica beads via ATRP as follows: NIPAAm (4.86 g, 42.9 mmol) was dissolved in 42.8 mL of 2-propanol in a flask at 1000 mM. To obtain a 1500-mM NIPAAm solution, NIPAAm (7.29 g, 64.4 mmol) was dissolved in 42.8 mL of 2-propanol. The NIPAAm solution was deoxygenated by bubbling with Ar gas for 30 min. CuCl (84.7 mg, 0.86 mmol), CuCl_2_ (11.5 mg, 0.086 mmol), and Me_6_TREN (220 mg, 0.959 mmol) were dissolved in NIPAAm monomer solution in an Ar atmosphere with stirring for 5 min, after which the flask was sealed and placed in a glove bag. Silane layer-modified silica beads (0.95 g) were placed in a glass vessel (50 mL), and the glass vessel containing the silica beads was placed in the glove bag. The oxygen in the glove bag containing the silica beads and monomer solution was removed by vacuuming and flowing Ar gas three times. Next, the monomer solution was poured into the silica beads in a glass vessel, and the glass vessel was sealed. This vessel was removed from the glove bag, and ATRP was performed for 16 h at 25 °C with continuous shaking of the reaction solution. Subsequently, the bead suspension was poured into two centrifuge tubes, to which acetone was added. The suspension containing the beads was centrifuged for 3 min at 1500 rpm, after which the supernatant was removed. A mixture of 50 mM ethylenediaminetetraacetic acid aqueous solution and methanol (v/v = 1:1) was added to the beads in the centrifuge tubes, and the suspension was sonicated for 30 min and centrifuged for 3 min at 1500 rpm. This procedure was repeated twice. The beads were then filtered, rinsed with pure water and acetone, and vacuum-dried for 2 h at 50 °C. All materials employed in the experiments are summarized in the Supplementary Material.

### Characterization of prepared PNIPAAm-modified beads

The prepared beads were characterized by CHN elemental analysis, FT–IR, and SEM to confirm immobilization of the silane layer and modification of PNIPAAm onto the silica beads.

The carbon composition of the silica beads was determined using an elemental analysis apparatus (PE-2400, PerkinElmer, Waltham, MA, USA), and the amount of silane layer was determined from the carbon composition of the beads, as shown in Eq. (1):

1 $$\frac{\%{C}_{S}}{\%{C}_{S} \left(calcd\right) \times \left(1 - \%{C}_{S}/\%{C}_{S}(calcd.)\right) \times S},$$

where *%C*_*S*_ is the increase in the carbon content of the beads after the silane coupling reaction, %*C*_*S*(*calcd.*)_ is the calculated carbon percentage of the mixed silane coupling reagent (CPTMS:GPTMS = 3:1), and *S* is the surface area of the silica beads (100 m^2^/g).

Further, the amount of PNIPAAm brush on the silica beads was obtained as follows:

2 $$\frac{\%{C}_{N}}{\%{C}_{N}\left(calcd\right) \times \left(1 - \%{C}_{N}/\%{C}_{N}\left(calcd.\right) - \%{C}_{S}/\%{C}_{S}(calcd)\right) \times S},$$

where *%C*_*N*_ is the increase in the carbon content of the beads after ATRP and %*C*_*N*(*calcd*)_ is the calculated carbon percentage of NIPAAm.

Modification of the polymer via ATRP was confirmed by observing the IR spectrum of the beads via ATR/FT–IR (FT/IR-4700; JASCO, Tokyo, Japan).

The morphology of the silica beads at each reaction step was observed using SEM (TM4000Plus-II, Hitachi High-Tech, Tokyo, Japan).

### HPLC analysis employing the prepared bead-packed column

The prepared beads were packed into two types of stainless steel columns (2.1 mm internal diameter × 50 mm length or 2.1 mm diameter × 100 mm length). The prepared PNIPAAm-modified beads were suspended in a methanol/water mixture (1:1, v/v). This suspension was poured into the reservoir of a column packer connected to an empty stainless steel column. The beads were packed by flowing a methanol/water mixture as the solvent at a constant pressure of 35 MPa for 60 min using an HPLC pump. After packing the beads, the column was connected to an HPLC system (Chromaster, Hitachi High-Tech Science, Tokyo, Japan), after which it was rinsed for 5 h at 40 °C under continuous flow of pure water at a flow rate of 0.2 mL/min.

Column performance was evaluated using hydrophobic steroids (the properties of the steroids are summarized in Supplementary Table S1). The steroids were dissolved in methanol at a concentration of 50 μg/mL and used as the sample. Pure water was used as the mobile phase at a flow rate of 0.2 mL/min, and the elution behavior of steroids was observed at a detection wavelength of 254 nm.

Thirteen types of drugs which require TDM were employed as samples to evaluate the applicability of the prepared column for TDM (drug properties are summarized in Supplementary Table S2). All drugs were dissolved in tetrahydrofuran at a concentration of 100 μg/mL. A CH_3_COONH_4_ solution (pH 6.75) was employed as the mobile phase at a flow rate of 0.2 mL/min, and the elution behavior of these analytes was observed at a predetermined wavelength for each drug (Supplementary Table S2) using an ultraviolet detector.

A serum–drug sample was prepared as follows. Freeze-dried serum was purchased from Nissui Pharmaceutical (Tokyo, Japan). The serum was prepared by dissolving freeze-dried serum in water (3 mL). For theophylline, sotalol, and phenobarbital, the drugs were dissolved in pure water at 1.0 mg/mL, after which the solution was filtered with a syringe filter (pore diameter: 0.2 μm). The serum solution was added to the drug solution, and the drug concentration in the serum was adjusted to 100 μg/mL. The other drugs were dissolved in tetrahydrofuran at a concentration of 1.0 mg/mL, filtered using a syringe filter, and 0.10 mL of the drug solution was collected into a microtube. The tetrahydrofuran was evaporated by flowing nitrogen gas into the solution. Next, 1.0 mL of serum was added to the drug to adjust the drug concentration to 100 μg/mL. CH_3_COONH_4_ solution (pH 6.75) was used as the mobile phase. The elution behavior of the analytes was observed at predetermined wavelengths for each drug (Supplementary Table S2) using an ultraviolet detector. For two-dimensional HPLC, the column was switched at a predetermined time to remove serum proteins.

## Supplementary Information

Supplementary Information.

## Data Availability

The datasets generated during and/or analysed during the current study are available from the corresponding author on reasonable request.
