# Supplementary material for: Two-dimensional temperature-responsive chromatography using a poly(N-isopropylacrylamide) brush-modified stationary phase for effective therapeutic drug monitoring
Source: Sci Rep. 2022 Feb 16;12:2653. doi: 10.1038/s41598-022-06638-1 (PMC8850448; doi:10.1038/s41598-022-06638-1)
Supplement: Supplementary file 1 — Supplementary Information. [file 41598_2022_6638_MOESM1_ESM.docx]

Supplementary Information

**Two-dimensional temperature-responsive chromatography using a poly(*N*-isopropylacrylamide) brush-modified stationary phase for effective therapeutic drug monitoring**

*Kenichi Nagase*, So Inoue, Masakazu Inoue, and Hideko Kanazawa*

Faculty of Pharmacy, Keio University, 1-5-30 Shibakoen, Minato, Tokyo 105-8512, Japan

*Corresponding author: Phone: +81-3-5400-1378; E-mail: nagase-kn@pha.keio.ac.jp

**Materials**

*N*-Isopropylacrylamide (NIPAAm), purified by recrystallization from *n*-hexane, was supplied by KJ Chemicals (Tokyo, Japan). Methanol, hydrochloric acid, acetone, toluene, tris(2-aminoethyl)amine, copper(I) chloride, copper(II) chloride, 2-propanol, ammonium acetate, hydrocortisone, prednisolone, dexamethasone, hydrocortisone acetate, testosterone, acetic acid, carbamazepine, diazepam, nitrazepam, zonisamide, vancomycin, phenobarbital, voriconazole, theophylline, lidocaine, and sotalol were purchased from Fujifilm Wako Chemicals (Osaka, Japan). Tris[2-(dimethylamino)ethyl]amine (Me_6_TREN) was synthesized from tris(2-aminoethyl)amine. Further, 3-glycidyloxypropyltrimethoxysilane (GPTMS) was obtained from Tokyo Chemical Industry (Tokyo, Japan). ((Chloromethyl)phenylethyl)trimethoxysilane (CPTMS) was purchased from Gelest (Morrisville, PA, USA). Freeze-dried serum was obtained from Nissui Pharmaceutical Co. (Tokyo, Japan). Silica beads (diameter: 5 μm, pore diameter: 300 Å; 100 m^2^/g) were obtained from Macherey-Nagel (Düren, Germany). The stainless-steel columns (inner diameter: 2.1 mm, column lengths: 50 mm and 100 mm) were purchased from Nishio Kogyo (Tokyo, Japan).

**Supplementary Table S1.** Properties of hydrophobic steroids

| Compounds | Structure | Molecular weight | Log*P* ^a^ |
| --- | --- | --- | --- |
| Hydrocortisone | 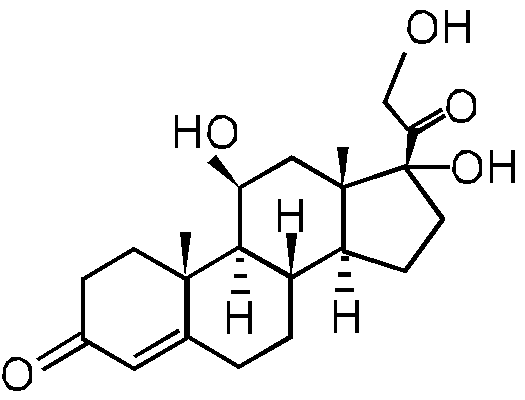 | 362.46 | 1.61 |
| Prednisolone | 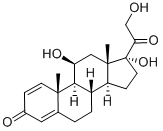 | 360.44 | 1.62 |
| Dexamethasone | 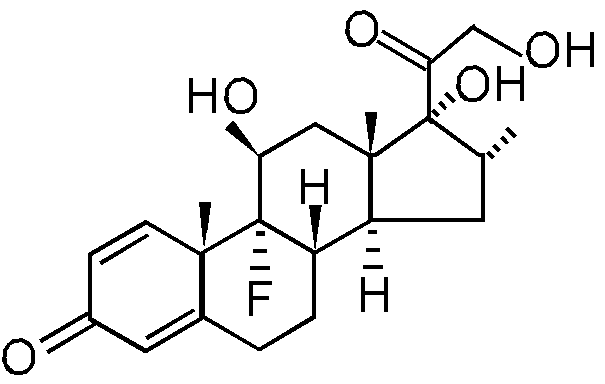 | 392.46 | 1.83 |
| Hydrocortisone acetate | 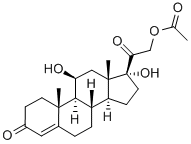 | 404.50 | 2.30 |
| Testosterone | 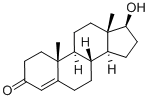 | 288.42 | 3.32 |

a) Partition coefficient of the *n*-octanol/water system


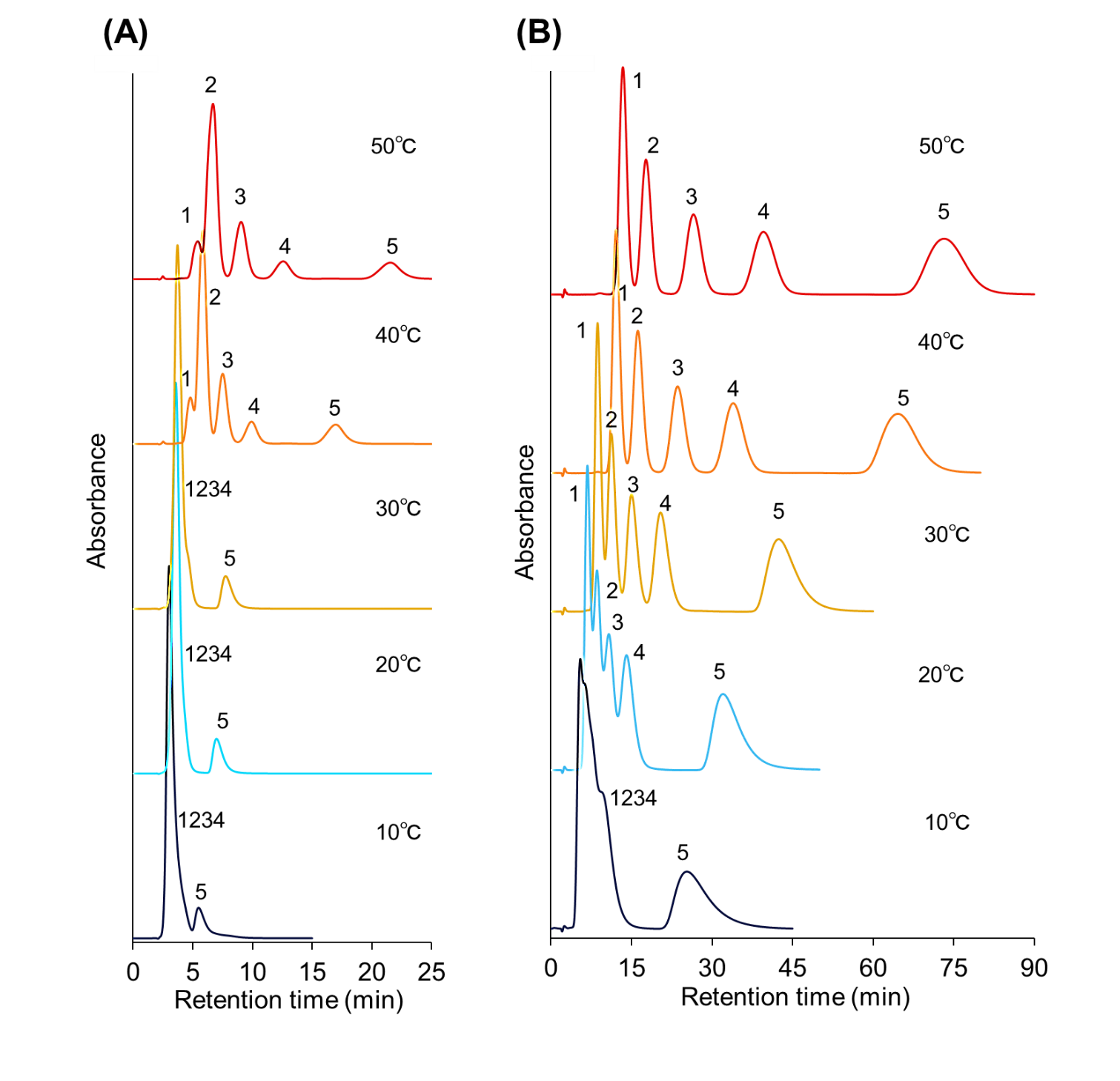


**Supplementary Fig. S1.** Chromatograms of hydrophobic steroids employing the PNIPAAm brush-modified bead-packed columns. (A) PN-1000 bead-packed column, (B) PN-1500 bead-packed column. The mobile phase was pure water, flow rate of the mobile phase was 0.2 mL/min, and detection wavelength was 254 nm. Peaks: 1, hydrocortisone; 2, prednisolone; 3, dexamethasone; 4, hydrocortisone acetate; and 5, testosterone.

**Supplementary Table S2.** Properties of drugs employed for therapeutic drug monitoring

| Classification | Compounds | Structure | Molecular weight | Log*P* ^a)^ | p*K*_a_ | Detection  (nm) |
| --- | --- | --- | --- | --- | --- | --- |
| Antiepileptic drug | Carbamazepine | 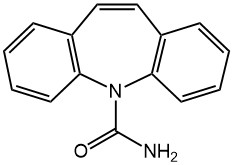 | 236.27 | 2.45 | 13.9 | 280 |
|  | Lamotrigine | 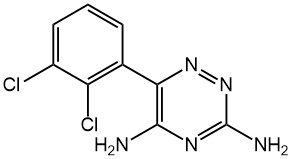 | 256.09 | 2.57 | 5.70 | 220 |
|  | Zonisamide | 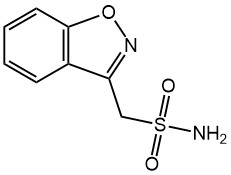 | 212.23 | 0.50 | 9.66 | 280 |
|  | Phenobarbital | 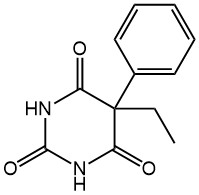 | 232.24 | 1.47 | 7.30 | 240 |
| Hypnotic | Nitrazepam | 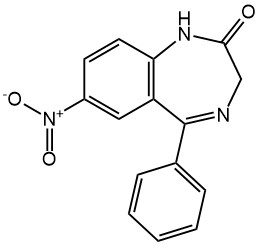 | 281.27 | 2.25 | 2.80 | 260 |
| Anxiolytic | Diazepam | 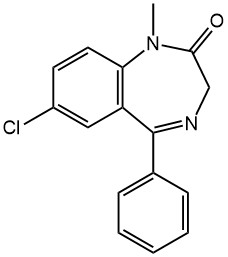 | 284.74 | 2.82 | 3.40 | 240 |

**Supplementary Table S2.** Properties of drugs in therapeutic drug monitoring.

| Classification | Compounds | Structure | Molecular weight | Log*P* ^a)^ | p*K*_a_ | Detection  (nm) |
| --- | --- | --- | --- | --- | --- | --- |
| Anti-arrhythmic drug | Disopyramide | 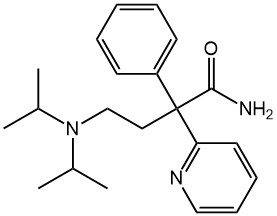 | 339.48 | 2.58 | 10.20 | 260 |
|  | Quinidine | 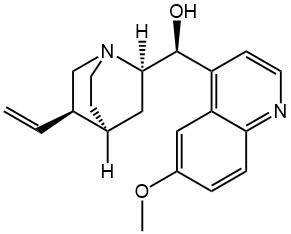 | 324.42 | 3.44 | 8.56 | 240 |
|  | Propafenone | 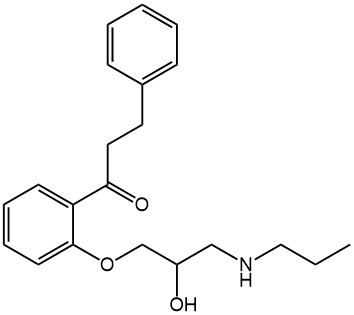 | 341.44 | 3.20 | 8.91 | 260 |
|  | Sotalol | 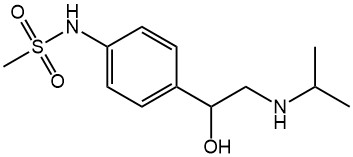 | 272.36 | 0.24 | 9.76 | 220 |
| Antifungal drug | Voriconazole | 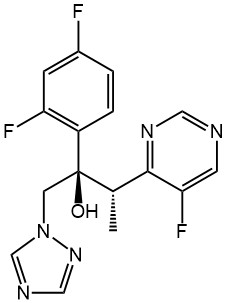 | 349.31 | 1.00 | 1.63 | 250 |
| Local anesthetic drug | Lidocaine | 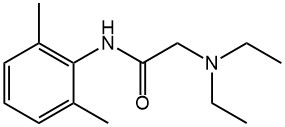 | 234.34 | 2.44 | 7.90 | 220 |
| Bronchodilator agent | Theophylline | 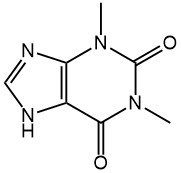 | 180.16 | −0.25 | 8.77 | 280 |

**Supplementary Table S3.** Drug retention times after three repeated measurements of each drug.

| Drugs | 10°C | | 20°C | | 30°C | | 40°C | |
| --- | --- | --- | --- | --- | --- | --- | --- | --- |
|  | RT (min) ^a)^ | RSD  (%) ^b)^ | RT (min) ^a)^ | RSD  (%) ^b)^ | RT (min) ^a)^ | RSD  (%) ^b)^ | RT (min) ^a)^ | RSD  (%) ^b)^ |
| Theophylline | 2.70 | 0.04 | 2.69 | 0.01 | 2.53 | 0.02 | 2.69 | 0.00 |
| Zonisamide | 2.50 | 0.09 | 2.79 | 0.01 | 2.82 | 0.01 | 2.98 | 0.01 |
| Voriconazole | 2.70 | 0.01 | 2.88 | 0.00 | 3.37 | 0.00 | 4.20 | 0.01 |
| Carbamazepine | 3.50 | 0.01 | 3.58 | 0.01 | 4.08 | 0.01 | 4.88 | 0.02 |
| Lamotrigine | 5.35 | 0.00 | 5.59 | 0.09 | 6.44 | 0.06 | 8.29 | 0.00 |
| Phenobarbital | 2.58 | 0.07 | 3.19 | 0.11 | 4.33 | 0.03 | 8.06 | 0.21 |
| Lidocaine | 3.53 | 0.00 | 3.60 | 0.00 | 3.84 | 0.02 | 4.30 | 0.01 |
| Quinidine | 2.08 | 0.00 | 2.14 | 0.00 | 2.21 | 0.05 | 2.28 | 0.01 |
| Disopyramide | 3.62 | 0.01 | 5.62 | 0.22 | 5.95 | 0.02 | 6.13 | 0.01 |
| Propafenone | 2.06 | 0.00 | 2.12 | 0.02 | 2.19 | 0.03 | 2.28 | 0.00 |
| Sotalol | 4.13 | 0.02 | 4.04 | 0.00 | 4.01 | 0.00 | 4.07 | 0.01 |
| Nitrazepam | 9.95 | 0.01 | 10.69 | 0.02 | 10.35 | 0.04 | 16.71 | 0.02 |
| Diazepam | 14.98 | 0.05 | 16.40 | 0.04 | 15.23 | 0.05 | 23.50 | 0.12 |

^a)^ Retention times of the drugs were measured and averaged from three separate measurements. b) Relative standard deviation was obtained by dividing the standard deviation of the retention time by the retention time and multiplying it.

**Supplementary Table S4.** Retention time of drugs in the serum after repeated measurement

| Drugs | Retention time (min) ^a)^ | RSD (%) ^b)^ |
| --- | --- | --- |
| Theophylline | 2.85 | 0.08 |
| Zonisamide | 5.83 | 0.08 |
| Voriconazole | 6.08 | 0.01 |
| Carbamazepine | 6.52 | 0.07 |
| Nitrazepam | 16.52 | 0.21 |
| Disopyramide | 4.54 | 0.20 |
| Diazepam | 23.35 | 0.06 |
| Phenobarbital | 7.01 | 0.08 |
| Lidocaine | 4.35 | 0.20 |
| Lamotrigine | 8.14 | 0.06 |

a) Retention times of the drugs were measured at 40°C using 10 mM CH_3_COONH_4_ buffer solution (pH 6.75) as the mobile phase and averaged after three separate measurements. b) Relative standard deviation was obtained by dividing the standard deviation of the retention time by the retention time and multiplying it.

**Supplementary Table S5.** Connection times of columns during two-dimensional high-performance liquid chromatography (HPLC)

| Drugs | Start of column connecting (min)^a)^ | End of column connecting (min) ^b)^ |
| --- | --- | --- |
| Carbamazepine | 5.00 | 8.00 |
| Voriconazole | 5.00 | 8.00 |
| Zonisamide | 4.50 | 7.00 |
| Lamotrigine | 6.50 | 9.00 |
| Diazepam | 21.0 | 26.0 |
| Nitrazepam | 15.0 | 18.0 |
| Phenobarbital | 6.00 | 10.0 |
| Disopyramide | 4.50 | 7.00 |

a) Start of connecting the primary column to secondary column. b) End of column connecting the primary column to the secondary column

**Supplementary Table S6.** Retention times of drugs analyzed using a two-dimensional HPLC system after repeated measurements

| Drugs | Retention time (min) ^a)^ | RSD (%) ^b)^ |
| --- | --- | --- |
| Zonisamide | 7.43 | 0.06 |
| Voriconazole | 15.17 | 0.13 |
| Carbamazepine | 16.32 | 0.07 |
| Nitrazepam | 25.68 | 0.01 |
| Disopyramide | 8.85 | 0.02 |
| Diazepam | 36.33 | 0.04 |
| Phenobarbital | 10.68 | 0.02 |
| Lamotrigine | 11.39 | 0.03 |

a) Retention times of the drugs were measured at 40°C using 10 mM CH_3_COONH_4_ buffer solution (pH 6.75) as the mobile phase and averaged after three separate measurements. b) Relative standard deviation was obtained by dividing the standard deviation of the retention time by the retention time and multiplying it.

**
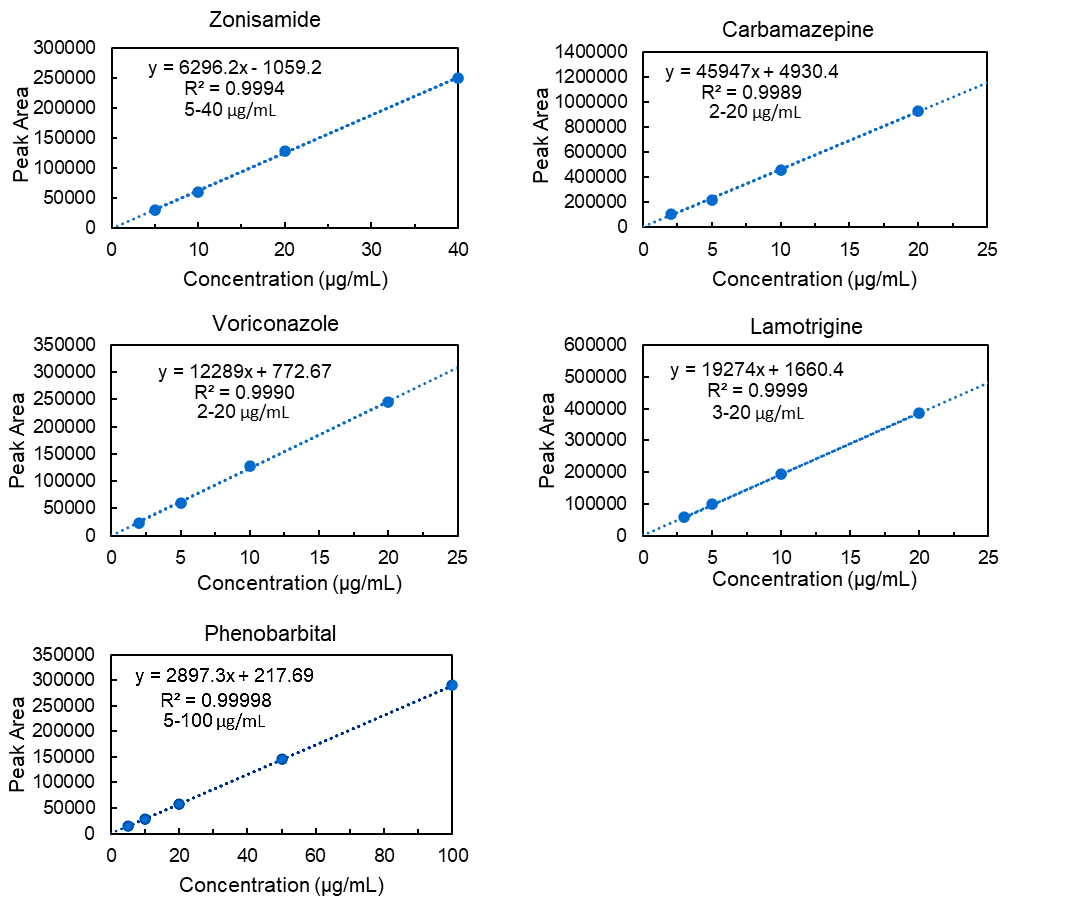
**

**Supplementary Fig. S2.** Calibration curve of drugs determined via temperature-responsive two-dimensional HPLC (n = 3)

**Supplementary Table S7.** Time of connecting the columns in two-dimensional HPLC

| Drugs | Start of column connecting (min)^a)^ | End of column connecting (min) ^b)^ |
| --- | --- | --- |
| Carbamazepine and Nitrazepam | 11.0 | 30.0 |
| Zonisamide and Carbamazepine | 5.50 | 15.0 |
| Lamotrigine and Nitrazepam | 8.50 | 50.0 |
| Lamotrigine and Diazepam | 8.00 | 35.0 |
| Zonisamide and Lamotrigine | 5.50 | 13.0 |

a) Start of connecting the primary column to secondary column. b) End of column connecting the primary column to the secondary column

**Supplementary Table S8.** Retention time of drugs analyzed using a two-dimensional HPLC system with repeated measurements

| Drugs | 1^st^ peak | | 2^nd^ peak | |
| --- | --- | --- | --- | --- |
|  | RT (min) ^a)^ | RSD (%) ^b)^ | RT (min) ^a)^ | RSD (%) ^b)^ |
| Carbamazepine and Nitrazepam | 12.97 | 0.13 | 28.36 | 0.39 |
| Zonisamide and Carbamazepine | 7.10 | 0.06 | 14.32 | 0.14 |
| Lamotrigine and Nitrazepam | 15.40 | 0.05 | 36.90 | 0.15 |
| Lamotrigine and Diazepam | 16.24 | 0.07 | 24.07 | 0.28 |
| Zonisamide and Lamotrigine | 7.43 | 0.03 | 13.89 | 0.08 |

a)Retention times of the drugs were measured at 40°C using 10 mM CH_3_COONH_4_ buffer solution (pH 6.75) as the mobile phase and averaged after three separate measurements. b) Relative standard deviation was obtained by dividing the standard deviation of the retention time by the retention time and multiplying it.
